# Harmony Search Algorithm for Word Sense Disambiguation

**DOI:** 10.1371/journal.pone.0136614

**Published:** 2015-09-30

**Authors:** Saad Adnan Abed, Sabrina Tiun, Nazlia Omar

**Affiliations:** Knowledge Technology Research Group (KT), Centre for Artificial Intelligent (CAIT), Universiti Kebangsaan Malaysia, 43600 UKM Bangi, Selangor, Malaysia; Beihang University, CHINA

## Abstract

Word Sense Disambiguation (WSD) is the task of determining which sense of an ambiguous word (word with multiple meanings) is chosen in a particular use of that word, by considering its context. A sentence is considered ambiguous if it contains ambiguous word(s). Practically, any sentence that has been classified as ambiguous usually has multiple interpretations, but just one of them presents the correct interpretation. We propose an unsupervised method that exploits knowledge based approaches for word sense disambiguation using Harmony Search Algorithm (HSA) based on a Stanford dependencies generator (HSDG). The role of the dependency generator is to parse sentences to obtain their dependency relations. Whereas, the goal of using the HSA is to maximize the overall semantic similarity of the set of parsed words. HSA invokes a combination of semantic similarity and relatedness measurements, i.e., Jiang and Conrath (jcn) and an adapted Lesk algorithm, to perform the HSA fitness function. Our proposed method was experimented on benchmark datasets, which yielded results comparable to the state-of-the-art WSD methods. In order to evaluate the effectiveness of the dependency generator, we perform the same methodology without the parser, but with a window of words. The empirical results demonstrate that the proposed method is able to produce effective solutions for most instances of the datasets used.

## Introduction

The most common problem of some Natural language processing (NLP) applications such as information retrieval and machine translation, is text ambiguity; which is a property of some English text. This problem is considered trivial, if the text is scanned by humans as they have the ability to identify the proper meaning of words that have multiple meanings, based on the context of that words. Humans acquired numerous amount of linguistic knowledge which enables them to judge precisely on the meaning of an ambiguous word. In the same manner as human judgement, a machine can address the ambiguity problem using the context of the target word and information about each meaning of that word. The context of the target word is extracted from the given text, while information about the target word′s meanings can be gained from external knowledge resources. These resources are divided into two main categories, as follows:

**Sense inventory resource.** In which structured information is provided about all senses (meanings) of each word. The information is the relationships among the words (thesauri), words glosses and examples (dictionaries), or taxonomies, and a set of semantic relationships (ontologies).
**Corpora resource.** This type provides collocation occurrences of a target word that cannot be gained from sense inventory resources. Corpora are either annotated or unannotated. Annotated corpora have been exploited in a classification manner [1–3]. Annotated corpus methods encounter an important problem, known as the knowledge acquisition bottleneck (manual sense-tagging). This problem was addressed by an automatic word sense discrimination method [4], which is based on unannotated corpus. This method induces word senses from the unannotated corpus by clustering word occurrences, and then classifying new occurrences into one of induced senses.

In the context of sense inventories, one of the earliest methods that exploit the use of sense inventory was conducted to perform WSD for the machine translation task, which is developed by Masterman [5]. In this method, the senses of a word are represented using the headings of the categories from Roget′s thesaurus [6] and the heading of the words that were most prominent in the context is selected. The use of machine readable dictionaries was fruitful when used by Lesk [7] to resolve word senses based on the sense definitions in the Oxford Advanced Learner′s Dictionary of Current English (OALD). The basic form of the Lesk algorithm disambiguates two words by finding the highest overlap among their sense definitions. The definitions of word senses tend to be fairly short and do not equip enough vocabulary to make fine-grained distinctions. Therefore, the expansion of sense definitions to accommodate the definitions of senses that are known as related to the senses being compared was an effective solution [8]. However, the Lesk algorithm leads to a combinatorial explosion of potential word sense combinations, when more than two words are considered. This problem was addressed by a variant of the Lesk algorithm that was based on a meta-heuristic search algorithm, which is simulated annealing to find the best sense combinations [9].

By adopting [9], various meta-heuristic algorithms were investigated for WSD, i.e., genetic algorithm [10–12], simulated annealing [13], and ant colony algorithm [14, 15]. These methods posed a movement from the use of the brute force approach [16], which is an intensive manner of all words in a given text being required to be disambiguated. However, the meta-heuristic methods for WSD mentioned can be seen in two groups, namely solo solution-based methods [9, 13] and population-based methods [10–12, 14, 15]. The latter try to explore more sense combinations in each iteration. Therefore, the multi-solutions methods are more promising at finding optimal (or near to optimal) sense combinations. These methods generally attempt to find the best sense combination that is coherent semantically.

The strength of the population based algorithm which is to explore more sense combinations, has motivated us to propose an evolutionary population-based method, known as Harmony Search Algorithm (HSA), to address the word sense disambiguation problem. HSA was developed by Geem et al. [17] to imitate the process of musical improvisation for the sake of finding harmonized musical notes. In comparison to conventional optimization algorithms, HSA has several characteristics that motivate our investigation in solving WSD. These characteristics are (i) HSA uses stochastic random searches. (ii) HSA has fewer parameters that need to be tuned. These parameters do not require a lot of tuning effort to attain a high quality solution [18]. (iii) HSA constructs a new solution by considering all existing solutions. This allows HSA to overcome the drawback of the building block theory of GA; whereby GA considers only two solutions in reproduction [18].

In order to evaluate each solution in the harmony memory of HSA, we used hybridized semantic similarity and related methods, i.e., Jiang and Conrath (jcn) [19] and an adapted Lesk algorithm [8]. The similarity measuring method (jcn) is capable of measuring the similarity for pairs of nouns and verbs parts-of-speech only. Therefore, a relatedness method, i.e., an adapted Lesk algorithm was used to work alongside the similarity method. These methods were selected based on the finding of Pedersen et al. [16], where they found that the adapted Lesk algorithm and jcn are the most accurate similarity and relatedness methods.

Aside from proposing HSA for WSD, we investigate the effectiveness of grammatical dependencies representations for WSD. The earliest use of dependency relations for WSD was proposed by Lin [20] when it was applied to syntactic dependencies to resolve word sense ambiguity. Practically, syntactic dependencies can be produced for each sentence separately. Hence, the context of the word being disambiguated will be determined from the sentence only, whereby the verbs are the most beneficiary from syntactic relations. However, nouns need for local collocations and wide context to be disambiguated [21]. Therefore, a generator of dependency parses is proposed in this work to identify the typed dependencies for the sentence being disambiguated beside the collocations from the neighbour sentences. For the purpose of proofing the efficiency of the dependency parser, we evaluated our method without the parser. The proposed method was experimented on benchmark datasets, namely SemCor [22] and Senseval-2 [23].

This paper is organized into five sections, including the current section, which introduced the proposed method and reviewed the related works. Section 2 presents a description of the WSD task. The proposed algorithm (HSDG) is presented in Section 3. A description and discussion of the experimental results are given in Section 4. Finally, Section 5 concludes the proposed method.

## Problem Description: Word Sense Disambiguation (WSD)

Word sense disambiguation is the problem of allocating the proper sense for an ambiguous word in a particular context. There are two variants of the WSD problem which are (i) Lexical sample; in which there is a specific set of words to be disambiguated. The most typical solutions for this type of WSD are the supervised classification methods. These methods are trained on a set of annotated instances and then implemented on a set of unannotated examples. The reason for the eligibility of these methods is the availability of annotated instances; as long as the lexical sample determines WSD for set of words only. (ii) All-words; this type of WSD intends to disambiguate all words in a given text that belong to any part-of-speech, i.e., nouns, verbs, adjectives, or adverbs. Based on the objective of this type, the supervised approaches encounter an acquisition bottleneck problem (manual sense-tagging). Meanwhile, unsupervised and knowledge-based approaches usually rely on full-coverage resources. Therefore, these approaches are the most eligible solution for all-words WSD.

In this study, we attempt to address the WSD of the all-words type using an automatic method that does not rely on annotated data. WSD can be seen as an optimization problem, whereas the words senses represent the solution variables, and the similarity and relatedness measurements between a pair of senses represents the fitness function that evaluates the quality of each solution. However, the difficulty of WSD varies based on the type of word senses. These can be categorized into two types as follows:

- Coarse-grained (or homograph): In this type, the senses of the ambiguous words can be clearly distinguished. For instance, consider the following two senses of the word “bank”—“sloping land beside a river” and “financial institution”. These two senses hold completely different meanings.
- Fine-grained (or polysemous): This type includes those senses that hold subtle differences. An example of fine-grained senses is the following two senses of the word “bank”—“financial institution” and “bank building”. The subtle differences between these two senses makes the task of WSD more complicated than it is in coarse-grained.

Several methods have been introduced to measure the semantic similarity or relatedness between a pair of senses that belong to both mentioned types. The semantic similarity measures are based on semantic networks that are defined in the WordNet [24]. These measures are either based on the notion of the path length [25] or based on the notion of information content [26] in a specific hierarchy (Eqs 1 and 2).

$$\begin{array}{c} Sim\left(C_{1},C_{2}\right)=1/L\left(C_{1},C_{2}\right) \end{array}$$ (1)

Where, *C*_1_ stands for concept 1 which represents sense 1, and L stands for the length from concept 1 to concept 2.

$$\begin{array}{c} Sim\left(C_{1},C_{2}\right)=IC\left(LCS\left(C_{1},C_{2}\right)\right) \end{array}$$ (2)

Where, IC refers to the information content, and LCS stands to the lowest common subsumer. LCS is the lowest concept in the WordNet hierarchy that subsumes the two concepts being measured.

Various measures are based on path length and information content notions [19, 27–30]. Most of these measures are capable of measuring the similarity for the concepts belonging to noun or verb parts-of-speech only, apart from [29], which is able to measure the similarity all parts-of-speech. Meanwhile, relatedness methods do not depend on the WordNet hierarchies or parts-of-speech. It measures the relatedness for any pair of concepts. The basic form of this measure quantifies the relatedness between two concepts by scoring the overlaps between their glosses [7], as illustrated in Eq 3.

$$\begin{array}{c} Relatedness\left(C_{1},C_{2}\right)=Gloss\left(C_{1}\right)\cap Gloss\left(C_{2}\right) \end{array}$$ (3)

## Proposed Method

**Fig 1:**
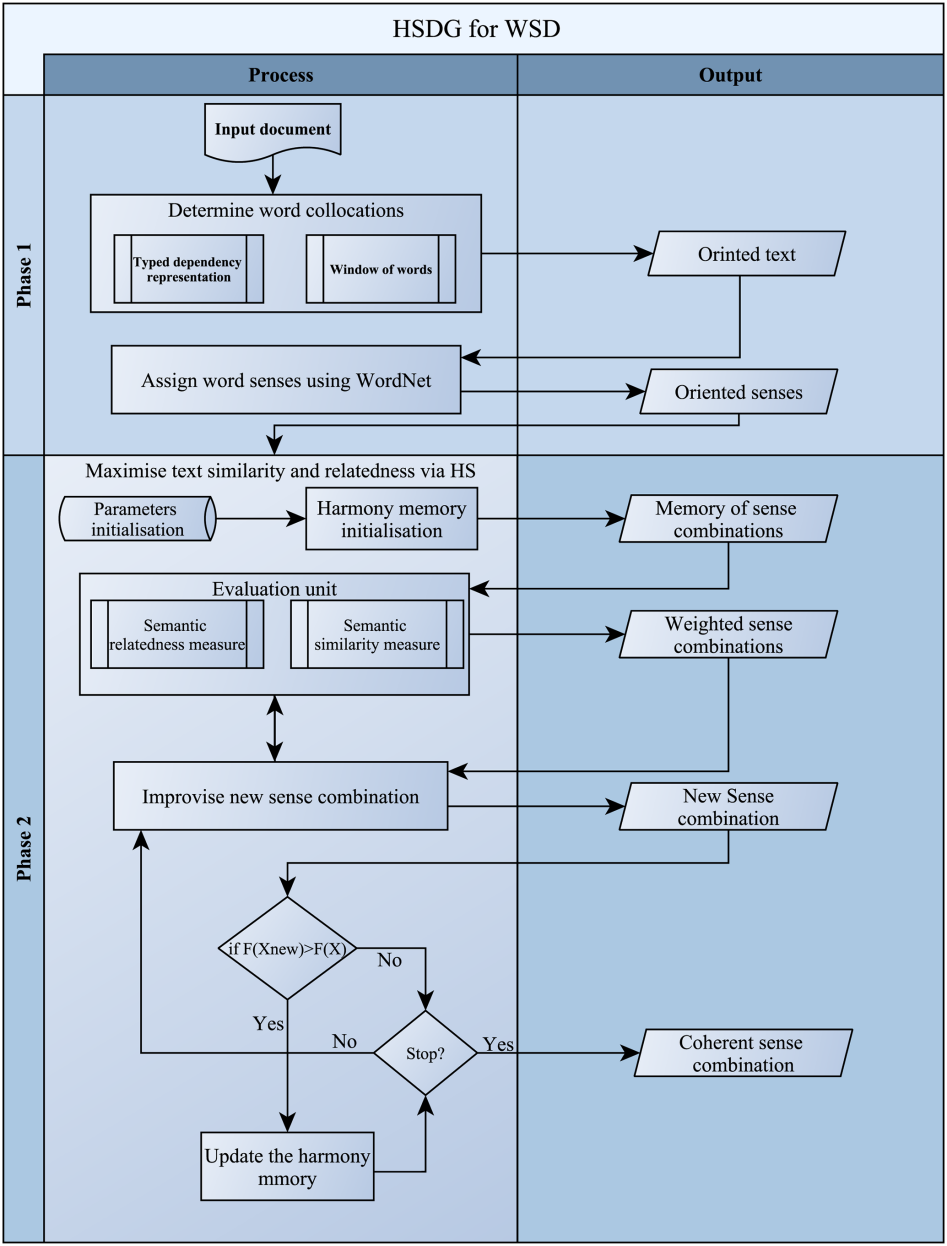
Methodology flowchart. Harmony search algorithm using dependency types and window of words for WSD.

The proposed method goes through two main phases (as shown in Fig 1). The first phase is the context extraction, which is conducted via the Stanford dependencies representation [31], and window of words determination. Meanwhile, the second phase is the process of maximising overall coherence (similarity or relatedness) via the Harmony Search Algorithm (HSA) [17]. Hence, this section presents a detailed description about the system of the dependencies representation. The harmony search algorithm, with its objective function for word sense disambiguation, will also be presented in this section.

### Dependencies generator for WSD

The first step in our methodology is to identify the collocation words of the words being disambiguated. This process was conducted through exploiting the typed dependencies of the given text, as well as, using a window of neighbouring words. Because some sentences are short, relying only on the typed dependencies, limits determining the collocation words. Consequently, this causes an inaccurate identification of the proper sense of the words being disambiguated. The use of a window of words in such situations helps us to overcome this limitation.

In the context of typed dependencies, we use the Stanford dependencies generator [31], which is a system that works on extracting the dependency parses of a sentence from the phrase structure parses and labels them with grammatical relations such as *object* and *subject*.

**Fig 2:**
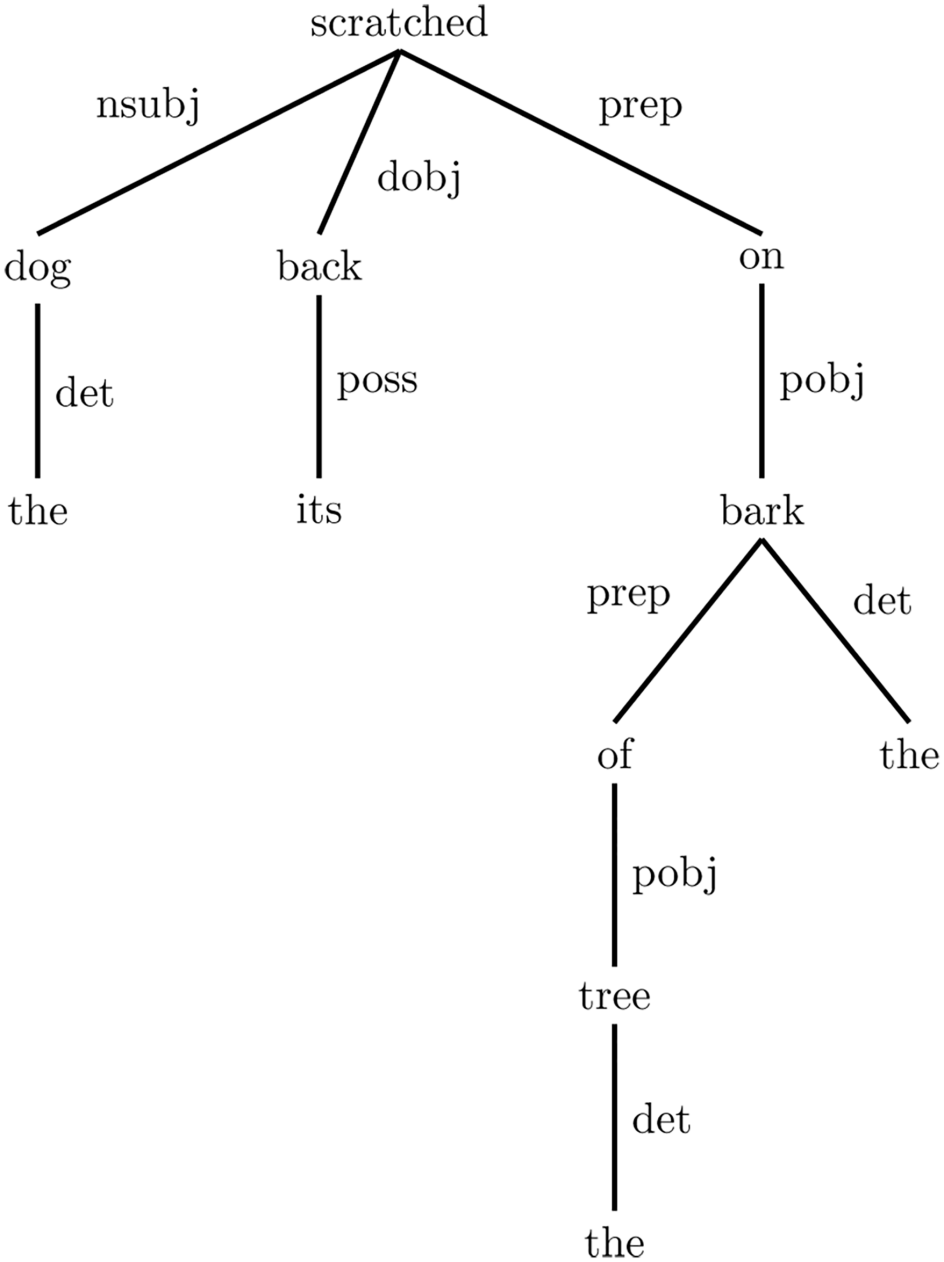
An example of generating typed dependencies. “The dog scratched its back on the bark of the tree”.

Practically, the process of generating typed dependencies has two phases. The first phase is dependency extraction; where the Stanford parser [32] is used to parse the sentence. The head of each part of the sentence is then identified, which is the semantic head of the part, rather than the syntactic head. This method of generating typed dependencies prefers the heads to be the content words, and have auxiliaries, complementisers, etc., depend on them. The second phase is dependency typing; which labels the extracted pair (head and dependent words) with a grammatical relation. For the purpose of illustrating the head and dependent words, in the context of generating typed dependencies, we pass an example sentence “The dog scratched its back on the bark of the tree” to the Stanford dependencies generator [31] (as shown in Fig 2).

Fig 2 shows a tree of the typed dependencies, where the head words are represented at the top edge and the dependent words appear at the bottom. The labels on the trees edges imply the grammatical relation title between the head and the dependent words. These labels are abbreviated, where the entire name of each of these relations is as follows (refer to [31] for reviewing all grammatical relations):

- nsubj: nominal subject
- dpbj: direct object
- pobj: object of preposition
- poss: possession modifier
- det: determiner
- prep: prepositional modifier

For the given example sentence, the preposition words can express a grammatical relation by themselves. Therefore, the Stanford dependencies generator provides the facility of collapsing multiple pairs of typed dependencies into a single pair, which then converges the words that are grammatically related. In consequence, it directs the similarity and relatedness methods (that are used in this study) to quantify the semantic amount in the collapsed pairs. The collapsed form of the given example is shown in Fig 2.

**Fig 3:**
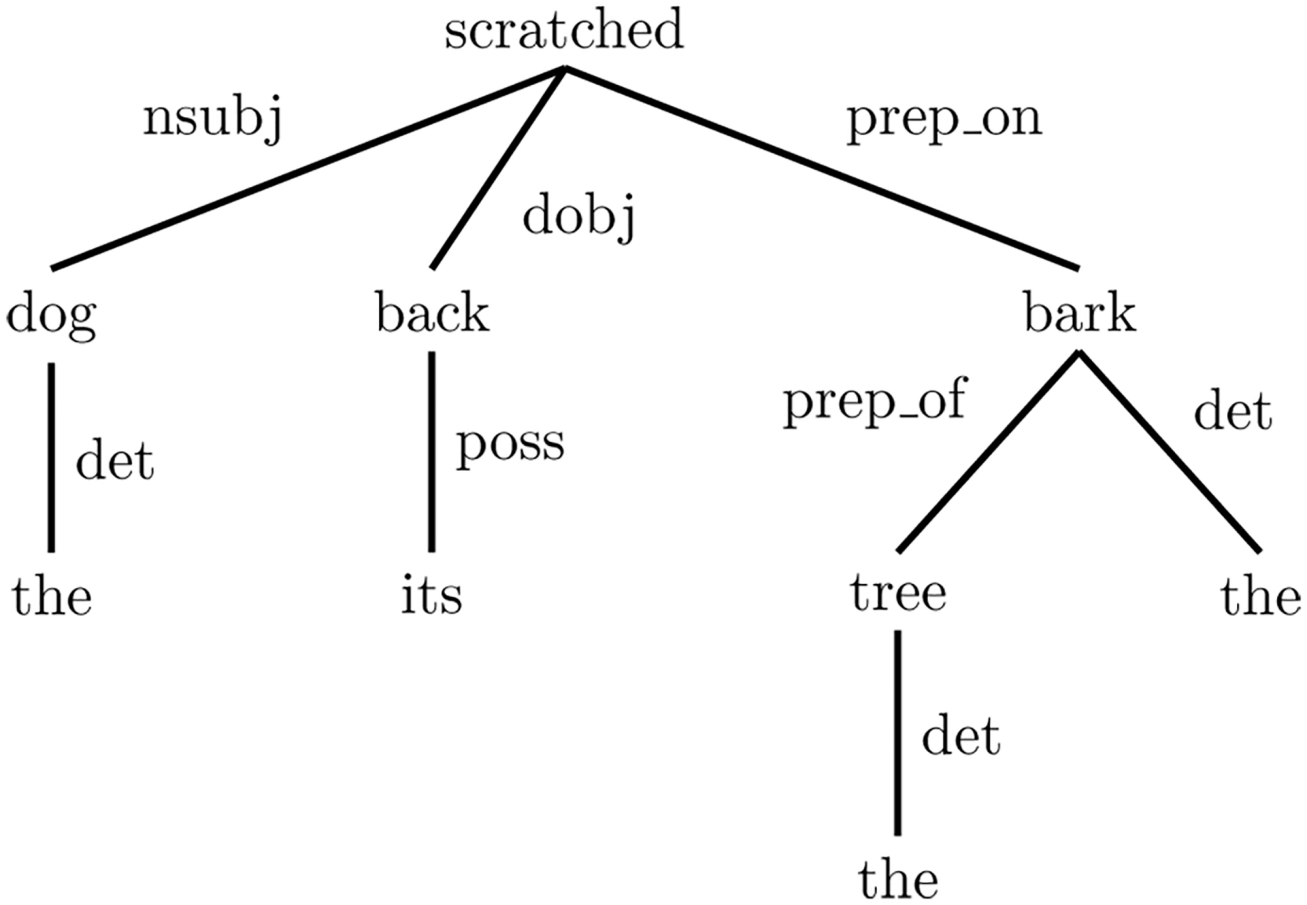
The collapsed form of the dependecy parses. “The dog scratched its back on the bark of the tree”

Obviously, the collapsed form of the dependencies shown in Fig 3 did shrink the number of stop words, such as prepositions words in the given example, with preservation of the grammatical structure of the sentence. Despite this, the collapsed form still carries useless grammatical relations in semantic measures, such as “det” and “root” in the given example. The proposed HSDG therefore neglects any of these useless relations; as they cannot be quantified semantically. However, the typed dependencies facilitate the task of disambiguating the sentence, where the ambiguous words in the sentence are disambiguated based on its heading or dependent word. This leads to excluding grammatically irrelevant words, which results in the noiseless substance of the sentence. For the purpose of explaining this process, we consider the word “bark” in the example shown in Fig 2. In the example, “bark” has two candidate senses based on the sentence context, which are “tough protective covering of the woody stems and roots of trees and other woody plants” and “the sound made by a dog”. The presence of the word “dog” in this example may mislead a WSD process in identifying the sense of “bark”. Hence, the significance of typed dependencies is obvious in directing the WSD system to choose the first sense of “bark” without confusion with the other sense.

### Harmony Search Algorithm for WSD

HSA is a stochastic evolutionary meta-heuristic algorithm, inspired from an artificial phenomenon, which is musical harmony [17]. The harmony of music achieved when the musicians play a combination of pleasing notes which are assessed by aesthetic criteria. In HSA, each decision variable gains a value corresponding to a musician who plays a note. A set of those variables form the solution that will be evaluated by the objective function that corresponds to the aesthetic criteria in music. The solution in HSA will be improved iteratively based on the candidate solutions from the harmony memory. In our proposed method, HSA attempts to find the best set of word senses that hold the maximum relatedness or similarity ratio. The HSA has four parameters that need to be initialized to specific values in order to control the process of finding the best solution. These parameters are as follows:

- Harmony Memory Size (HMS): is the number of harmonies (solutions) in the harmony memory. In this work, the maximum number of senses held by a word in a sentence will be assigned to an HMS that does not exceed twenty, because senses beyond twenty rarely occur. Consequently, the harmony memory of HSA will hold all potential senses for each word in the given text.
- Harmony Memory Consideration Rate (HMCR). This parameter indicates the rate of selecting value from the harmony memory. The value of this parameter varies from 0.7 to 0.99. In this methodology, the assigned value of HMCR is 0.95, in order to increase the chance of constituting a new solution from the old memory.
- Pitch Adjust Rate (PAR). This parameter indicates the rate of modifying the value (that has been selected from the harmony memory) to one of the neighbouring values. The modification of the value in this methodology is either by one increment or one decrement, with respect to the allowed range of the solution variable. The value of this parameter varies from 0.1 to 0.5. In this work, the assigned value of PAR is 0.25.

**Fig 4:**
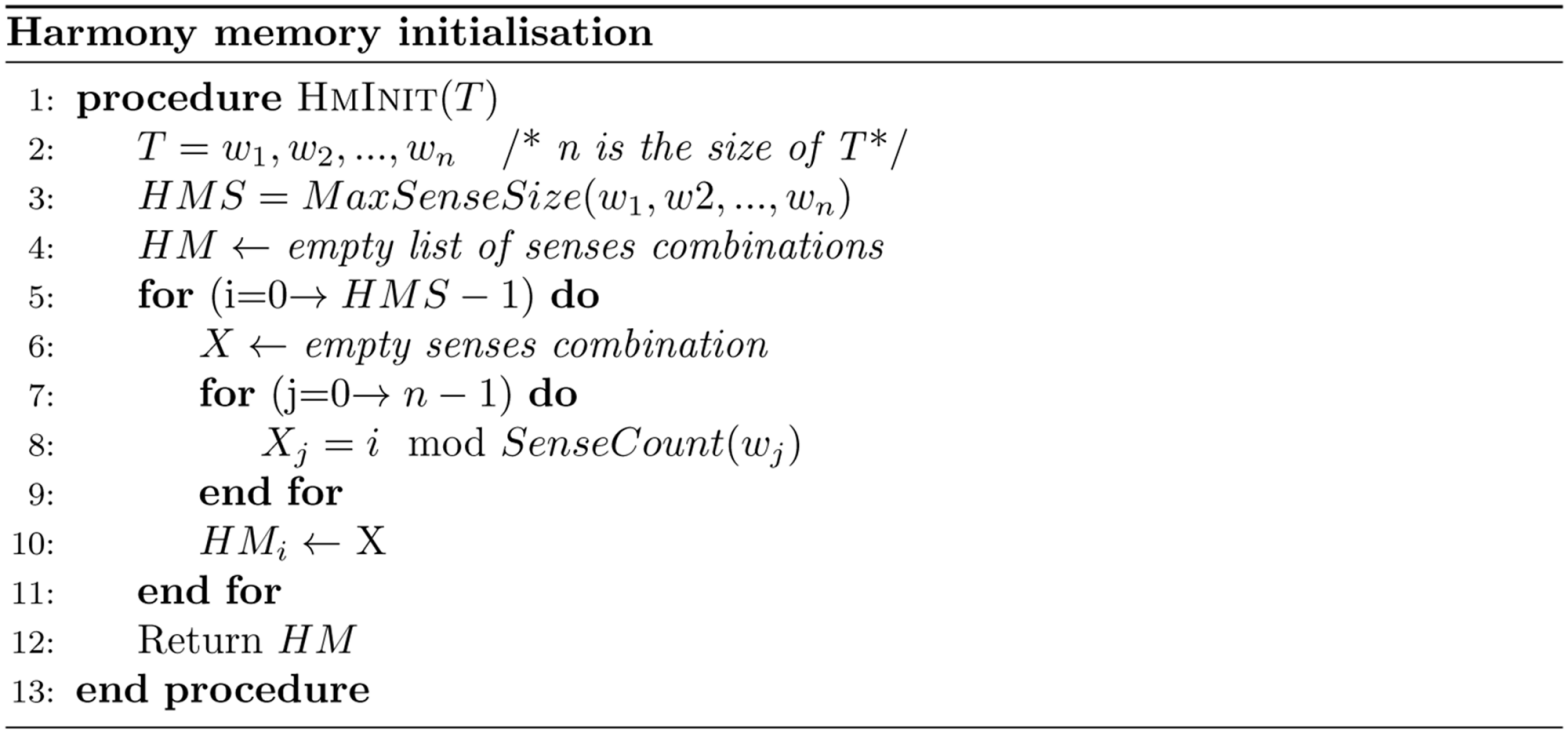
Harmony memory initialisation. The pseudo code of initialising the harmony memory of HSA for WSD.

**Fig 5:**
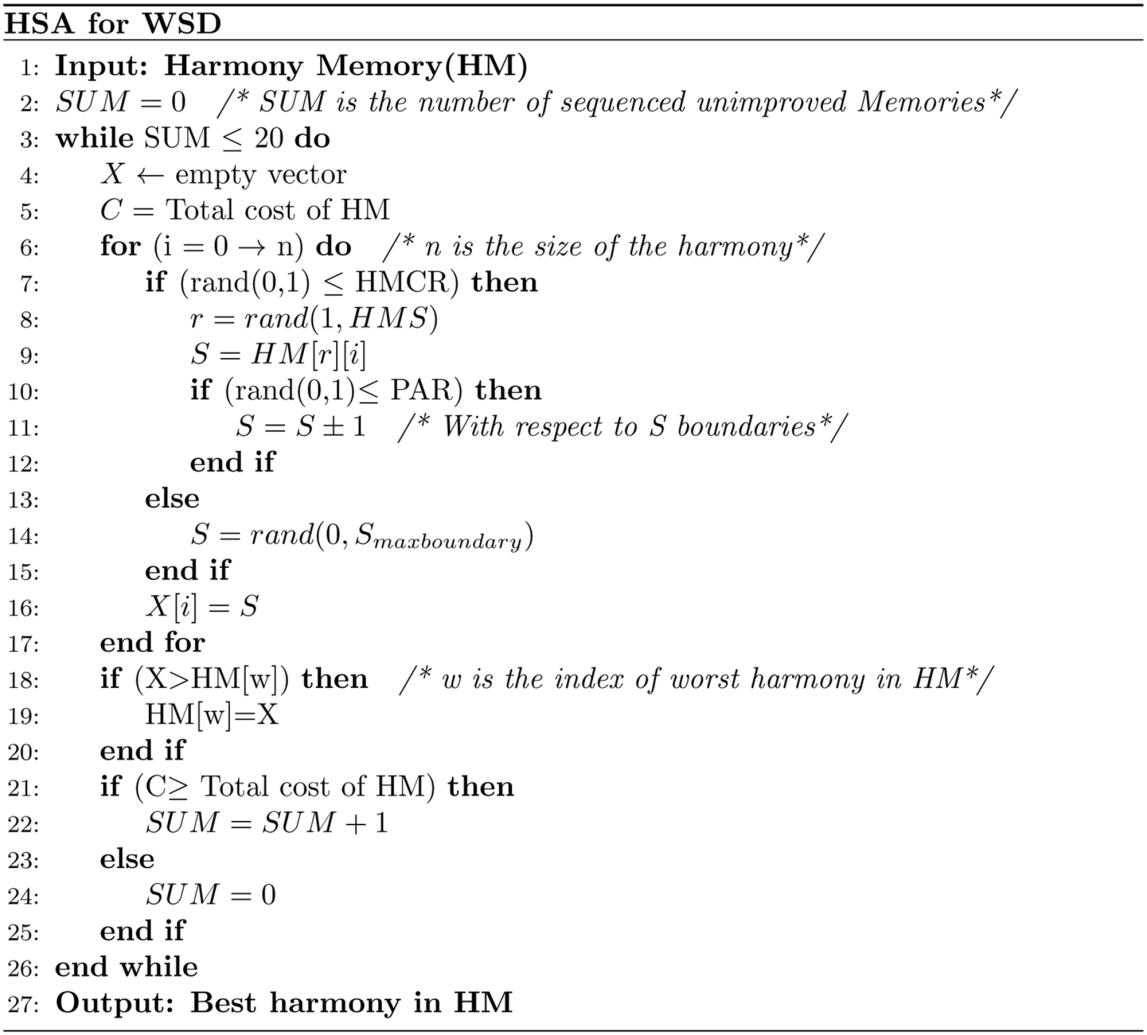
HSA for WSD. The main steps of finding the best senses combination for an instance of WSD problem using HSA.

After initialising the mentioned parameters, the HSA process carries out the following four steps as in [17]:

- Step 1: Initialise the Harmony Memory (HM). This step initialises the HM with a number of solutions (sets of senses) with respect to the HMS value. Due to the necessity of covering all senses for each word, we initialised the HM by the pseudo code shown in Fig 4. Moreover, the harmony memory holds a solution that presents the most frequent sense for each word to improve the successors solutions without being the final solution.
- Step 2: Improvise a new harmony (senses combination). This is the process of combining a new solution from the HM within the HMCR value, or randomly within 1-HMCR value. Any part (word sense) of the new solution selected from the HM will be adjusted within PAR value. This adjustment is represented by increasing or decreasing the selected value (word sense) by one with respect to the boundaries of the selected value. The improvisation process of senses combination presented in Fig 5 from line 6 to line 17.
- Step 3: Update the HM. This step replaces the worst solution in the harmony memory with the improvised solution; if the latter has a better fitness value. The fitness value of the solution is calculated by the objective function which will be explained in the next subsection (objective function of HSA). The update step is shown in Fig 5 from line 18 to line 20.
- Step 4: Repeat steps 2 and 3 until the stopping criterion is met. In this work, HSA stops after twenty sequenced and unimproved memories, which has been been determined experimentally. This step presents the main loop of HSA which is shown in line 3 of Fig 5

#### Evaluation unit: the objective function of HSA

The objective function is the criteria that determine the quality of each solution in the harmony memory. In our proposed method, the objective function is presented as a combination of semantic similarity and relatedness methods, namely Jiang and Conrath (jcn) method [19] and the adapted Lesk algorithm [8]. The objective function measures the similarity or the relatedness for each pair of concepts (solution variables) that hold a collocation relation or a dependency relation (this relation identified by the dependency generator). The pair of concepts that belong to noun or verb parts-of-speech is measured using jcn; otherwise adapted Lesk algorithm measures that pair. The modality of these measures is presented as follows:

**Semantic similarity measure.** This type of measure is used in a very particular sense; as it implies the correlation between two concepts, according to information founded in a specific hierarchy. Semantic similarity measures quantify the similarity between pairs of nouns or verbs only, because these measures rely on WordNet hierarchies, which do not mix between different parts-of-speech. However, there are two main variants of similarity measures, namely path length and information content. The path length measure counts the physical length between the concepts being measured based on a specific hierarchy. This measure seems inaccurate enough, since the path between extremely specific concepts (e.g., “pitchfork”), exhibits much smaller distinctions in semantic similarity than path lengths of extremely general concepts (e.g., “artifact”). Hence, this study exploits the use of information content method that records a higher score of similarity for very specific concepts and less for extremely general concepts.
  In general, the information content measure is defined as the negative logarithm of concept probability as illustrated in Eq 4. In the basic form of information content notion, the similarity score between two concepts is given as the information content value of the concept that subsumes the two concepts being measured based on a specific semantic hierarchy as presented in Eq 2 [26].
  $$\begin{array}{c} IC(C)=-\log P(C) \end{array}$$ (4)
  Where, C and IC are a concept and the information content of that concept respectively.
  In the Resnik method, the similarity calculation considers only the information content for the LCS concept of the concepts being measure (as shown in Eq 2). This poses an inaccurate similarity ratio since it is not comprehensive for all possible information contents for the concepts being measured. Hence, some methods attempt to exploit information content for both of the concepts being measured and their LCS concept. JCN is one of the methods that pays attention to the importance of the information content of the concepts being measured alongside the information content of the LCS of these concepts (as shown in Eq 5).
  $$\begin{array}{c} Dist_{jcn}\left(C_{1},C_{2}\right)=IC\left(C_{1}\right)+IC\left(C_{2}\right)-2\times IC\left(LCS\left(C_{1},C_{2}\right)\right) \end{array}$$ (5)
  The jcn method was used in this study as a member of the evaluation unit that is responsible for the assessment of sense combinations (solutions). As mentioned earlier, the similarity method able only to measure the similarity between identical pairs of nouns or verbs parts-of-speech. Therefore, this study invokes a relatedness measure to complement the measurement process for all parts-of-speech to fulfil the duty of the evaluation unit in the HSA. Moreover, the promising achievement that has been achieved by combining similarity and relatedness methods in [33], motivates us to investigate a combination of similarity and relatedness methods.
**Relatedness measures.** This type of measure is represented by the notion of gloss overlap [7], which is introduced as the number of common words between the glosses of two concepts (see Eq 3). The gloss overlap notion is more general than the similarity notion; since it measures the commonality between any two concepts, regardless of their parts-of-speech. The basic form of the relatedness measure is given in the Lesk algorithm, which measures the relatedness between two concepts by considering the overlaps between their glosses. The Lesk algorithm encounters poverty of information to make fine-grained distinctions, due to the shortness of the concept glosses. Banerjee and Pedersen [8] suggested to expand the gloss overlap method to accommodate the glosses of the concepts that are known as being related to the concepts being measured. The adapted Lesk algorithm takes two concepts as an input. While, the output is a numeric value that indicates the relatedness score between the inputted concepts, which is given in Eq 6.
  $$Relatedness\left(C_{1},C_{2}\right)=\sum score\left(R_{1}\left(C_{1}\right),R_{2}\left(C_{2}\right)\right)\forall \left(R1,R2\right)\in RELPAIRS$$ (6)
  Where, RELPAIRS represents a set of pairs of semantic relations. These pairs are defined in a reflexive relation as follows:
  $$RELPAIRS=\{\left(R_{1},R_{2}\right)|R_{1},R_{2}\in RELS;if\left(R_{1},R_{2}\right)\in RELPAIRS,then\left(R_{2},R_{1}\right)\in RELPAIRS\}$$ (7)
  Where, R1 and R2 stand for specific WordNet semantic relations, such as hypernymy, hyponymy, gloss, etc. These relations act as the REL part, while PAIRS is constituted from relating these relations together in the form of pairs as follows:
  $$Relatedness\left(C_{1},C_{2}\right)=score\left(hypo\left(C_{1}\right),hypo\left(C_{2}\right)\right)+score\left(hype\left(C_{1}\right),hype\left(C_{2}\right)\right)+score\left(gloss\left(C_{1}\right),gloss\left(C_{2}\right)\right)+score\left(gloss\left(C_{1}\right),hype\left(C_{2}\right)\right)+score\left(hype\left(C_{1}\right),gloss\left(C_{2}\right)\right)$$
  Obviously, these pairs satisfy to the reflexive attribute of RELPAIRS. This implies that the relatedness degree between C1 and C2 is indeed identical to the relatedness of C2 and C1. However, the adapted Lesk algorithm quantifies the relatedness degree for those concepts that belong to different parts-of-speech, as well as, for identical parts-of-speech that belong to adverbs or adjectives.

In this study, the adapted Lesk algorithm is responsible for measuring all pairs of concepts; unless those pairs belong to noun or verb parts-of-speech which are assigned to the jcn measure. Also, adapted Lesk algorithm measures the relatedness for the pairs that jcn fails to quantify their similarity. The reason of the jcn inability to measure the similarity for some noun and verb pairs is attributed to the absence of the occurrences record, in consequence, the information content for these concepts can not be estimated. However, the objective function that used in the HSA is a collaboration between jcn and adapted lesk measures.

## Experimental results

In this study, we present the use of HSA for WSD based on a combination of similarity and relatedness measures. These measures are oriented by a dependency generator that selects the words to be measured based on dependency types. This section presents and discusses the results obtained. The results include all experimental results, apart from those using the dependency generator to show the impact of the latter on the entire proposed method. Analogously, the obtained results in this study are comparable to the state-of-the-art methods that follow a similar approach. This was proven in a comparison of our results against other methods results, when tested on the same dataset. We compared the results based on the metrics defined in [34] as follows:

- **Coverage:** The measure of the method comprehensiveness which is the percentage of the answered instances to the overall instances:
  $$\begin{array}{c} \text{Coverage}=\frac{\text{all}\;\text{answered}\;\text{senses}}{\text{total}\;\text{of}\;\text{senses}} \end{array}$$ (8)
- **Precision:** The measure of the method correctness based on the number of answered instances:
  $$\begin{array}{c} \text{Precision}=\frac{\text{correct}\;\text{answered}\;\text{senses}}{\text{total}\;\text{of}\;\text{answered}\;\text{senses}} \end{array}$$ (9)
- **Recall:** The measure of the method correctness based on answered and abandoned instances:
  $$\begin{array}{c} \text{Recall}=\frac{\text{correct}\;\text{answered}\;\text{senses}}{\text{total}\;\text{of}\;\text{senses}} \end{array}$$ (10)
- **F-measure:** The harmonic average of precision and recall measures
  $$\begin{array}{c} F-\text{measure}=2\times \frac{Precision\times Recall}{Precision+Recall} \end{array}$$ (11)

The results obtained were evaluated based on two types of dataset, i.e., set of SemCor files [22] and Senseval-2 English all-words [23]. Specifically, we selected nineteen files from SemCor 3.0 files based on the literature, namely br-a01, b13, c0l, d02, e22, r05, g14, h21, j0l, k01, k11, l09, m02, n05, p07, r04, r06, r08, and r09. One of the aims of this study is to show the impact of dependency representations in selecting feature words. Hence, we show the results of HSA based on each window of words and the dependency relations (Table 1).

**Table 1:** Comparison between the dependency types and window of words based on recall metric.

	POS	Context Selection		
		Five words	Eleven words	Dependency types
**SemCor3.0**	**Noun**	68.30%	69.41%	65.47%
	**Verb**	43.70%	44.66%	43.66%
	**Adj.**	66.65%	67.88%	66.12%
	**Adv.**	60.24%	61.16%	59.36%
	**All**	60.21%	61.27%	58.79%
**Senseval-2**	**Noun**	67.51%	68.48%	68.31%
	**Verb**	38.72%	39.41%	38.89%
	**Adj.**	58.42%	60.39%	59.08%
	**Adv.**	57.19%	58.52%	57.49%
	**All**	57.82%	58.91%	58.39%

In the context of window of words, Table 1 shows an obvious improvement in the disambiguation results when the window of words was widened. This improvement was due to expanded the information that aided in disambiguating the words. However, selecting feature words according to window size is not satisfied to any semantic or syntactic principle, which may have invoked noisy words during disambiguation process. Therefore, our proposed method investigates the effect of the dependency relations of syntactic parsing, in terms of selecting the feature words; which is motivated by the effectiveness of the disambiguation methods in [20, 35]. These words form the main context that will be used to disambiguate the target word. Table 1 shows the obtained disambiguation results using the dependency relations. The dependency relations are the outcome of parsing the syntactic parsing operation, which is sentence based. This fact poses a limit on the size of the selected context. Consequently, the short sentences will not be disambiguated effectively because there context is very short. In Table 1, the dependency representations field shows an obvious refinement across the two datasets, i.e. SemCor 3.0 and Senseval-2. This is attributed to the length of the sentence, which in Senseval-2 is equal to 23 words on average. Meanwhile, in the selected files of SemCor 3.0 the average sentence length is 19 words. The dependency representations negatively affect the disambiguation result when sentences are short. We therefore attempted to refine the disambiguation process of short sentences that used dependency relations by combining them with window of words. This combination′s discipline is to not exceed eleven words, including dependency types (as shown in Table 2).

**Table 2:** The results obtained from combining the typed dependencies with window of words.

	**POS**	**Coverage**	**Precision**	**Recall**	**F-measure**
**SemCor3.0**	**Noun**	99.83%	72.19%	72.07%	72.12%
	**Verb**	82.39%	55.81%	45.98%	50.42%
	**Adj.**	100%	71.02%	71.02%	71.02%
	**Adv.**	100%	64.51%	64.51%	64.51%
	**All**	94.95%	67.03%	63.73%	65.33%
**Senseval-2**	**Noun**	97.97%	70.53%	69.10%	69.80%
	**Verb**	94.66%	42.54%	40.24%	41.35%
	**Adj.**	97.59%	62.55%	61.05%	61.79%
	**Adv.**	95.31%	62.80%	59.86%	61.29%
	**All**	96.80%	61.70%	59.72%	60.69%

**Fig 6:**
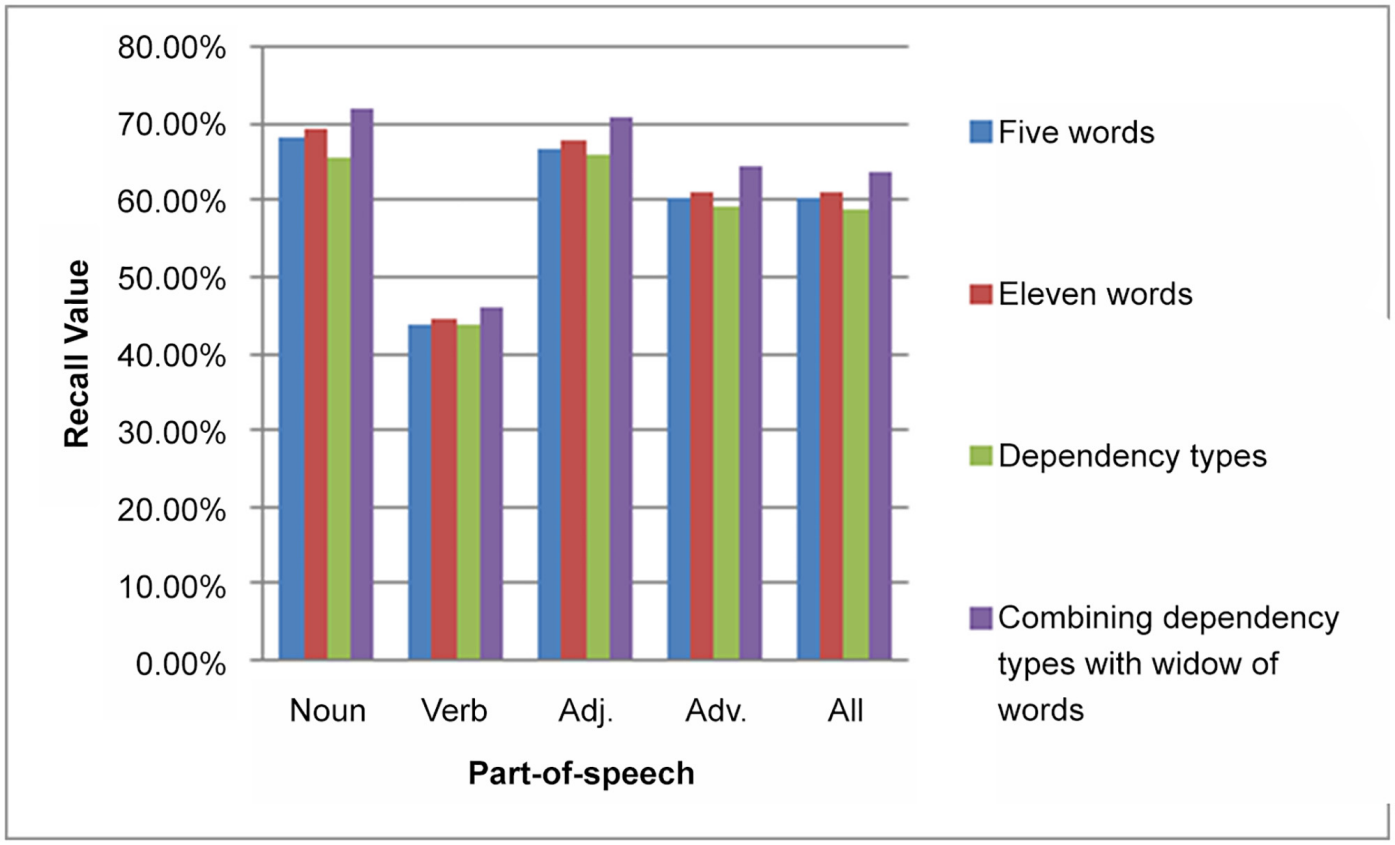
Context selection effectiveness. HSA efficiency using various context selection methods over the selected set of files from SemCor 3.0.

Combining the dependency types with window of words revealed an improvement over both datasets as seen in Table 2. This improvement was because the feature words of the dependency generator were complemented by the window of words. This window of words was obtained from the adjacent sentences, as the dependency generator only provides feature words from the current sentence, which includes the word being disambiguated. For comparison purposes, Fig 6 shows the HSA results obtained over different context selection methods.

### Comparison of HSA to related works

The proposed method in this study attempts to exploit knowledge based methods in an intelligent search technique known as HSA. Similar methods have been conducted to perform the task of WSD, as mentioned in Section 1. Table 3 compares the results obtained using HSA against two methods which are the genetic algorithm [11], and the WSD combining conceptual distance, frequency and gloss [36]. The latter is an improved version of conceptual density, this improvement known as Cluster Depth Correction (CDC).

**Table 3:** Comparison of HSA to related works based on noun part-of-speech over nineteen files from SemCor corpus.

Method	Coverage	Precision	Recall	F-measure
CDC	76.81%	**80.91%**	62.19%	70.32%
WGWSD	**100%**	71.96%	71.96%	71.96%
HSDG for WSD	99.83%	72.19%	**72.07%**	**72.12%**

Table 3 shows that Zhang et al. [11] achieved the task of WSD with full coverage over noun part-of-speech, which led to yield a precision value equivalent to that of the recall. Rosso et al. [36] obtained the highest precision value, but with the lowest coverage that led to lowest recall value. Meanwhile, Zhang et al. [11] and our method outperformed their recall value. In the context of recall, we have obtained a recall value slightly higher than Zhang et al. [11]. However, Zhang et al. leaned towards intensive knowledge-based assets, by involving the WordNet domains in their method.

Based on the Senseval-2 dataset, we compared the proposed HSDG with the genetic algorithm [12], ant colony algorithm [15], and the unsupervised graph based method [33] as shown in Table 4. Nguyen and Ock [15] presented the WSD problem in the form of Travelling Salesman Problem (TSP) to find the shortest path based on semantic relatedness. While, Hausman [12] exploited the use of a genetic algorithm to maximise the overall semantic similarity of the text. Apart from meta-heuristic search, Sinha and Mihalcea [33]combined variant graph algorithms in voting scheme to achieve a system that perform the task of WSD based on similarity and relatedness measures, i.e. jcn, lch [28] and Lesk.

**Table 4:** Comparison of HSA to related works based on the Senseval-2 dataset.

Method	Coverage	Precision	Recall	F-measure
**TSP-ACO**	99.70%	63.00%	62.80%	62.89%
**Genetic algorithm**	92.12%	49.73%	52.29%	50.79%
**HSDG for WSD**	96.80%	61.70%	59.72%	60.69%
**Voting graph centrality**	-	58.83%	56.37%	57.94%

In general, all methods shown in Table 4 unless the last one, attempt to maximise the relatedness or similarity of text based WordNet hierarchies and concepts glosses. While, the last method solves WSD in voting manner using PageRank, indegree, closeness and betweenness. The genetic algorithm in [12] exploited the use of various semantic relations in different combination schemas. The other methods shown in Table 4 make use of semantic similarity and relatedness measures, which lead to better accuracy in comparison to [12]. Obviously, TSP-ACO outperforms the other methods as it mapped the words to their glosses and then employed the graph centrality which would be searched using ACO. Finally, our method was comparable to each of the [33] and [15], despite it not using any graph centrality scheme, but rather, exploiting the use of dependency types.

## Conclusion

WSD for bag of words was dealt with as a combinatorial problem. Employing meta-heuristic search techniques was successfully solved, such as type of problems. The main aim of the work presented in this paper was to investigate the performance of Harmony Search Algorithm using different types of context selecting methods, i.e., window of words, dependency types, and hybridizing dependency types using window of words. We observed that the hybridized context selection method outperformed all the other employed methods. The proposed algorithm was evaluated on different types of datasets from SemCor 3.0 and Senseval-2. Based on SemCor 3.0 and Senseval-2 evaluation, HSA using dependency types performed well, if the length of the sentence was adequate, i.e., each word in the sentence had dependent content words. However, the overall results demonstrate the effectiveness of the proposed method in solving WSD.
